# Long length of stay at the emergency department is mostly caused by organisational factors outside the influence of the emergency department: A root cause analysis

**DOI:** 10.1371/journal.pone.0202751

**Published:** 2018-09-14

**Authors:** Babiche E. J. M. Driesen, Bauke H. G. van Riet, Lisa Verkerk, H. Jaap Bonjer, Hanneke Merten, Prabath W. B. Nanayakkara

**Affiliations:** 1 Department of Emergency Medicine, VU University Medical Center, Amsterdam, The Netherlands; 2 VU University school of medical sciences, Amsterdam, the Netherlands; 3 Section Acute Medicine, Department of Internal Medicine, VU University Medical Center, Amsterdam, The Netherlands; 4 Department of Surgery, VU University Medical Center, Amsterdam, The Netherlands; 5 Department of Public and Occupational Health, Amsterdam Public Health research institute, VU University Medical Center, Amsterdam, The Netherlands; 6 Acute Care Network North-West, VU University Medical Center, Amsterdam, The Netherlands; Iran University of Medical Sciences, ISLAMIC REPUBLIC OF IRAN

## Abstract

**Background:**

Emergency department (ED) crowding is common and associated with increased costs and negative patient outcomes. The aim of this study was to conduct an in-depth analysis to identify the root causes of an ED length of stay (ED-LOS) of more than six hours.

**Methods:**

An observational retrospective record review study was conducted to analyse the causes for ED-LOS of more than six hours during a one-week period in an academic hospital in the Netherlands. Basic administrative data were collected for all visiting patients. A root cause analysis was conducted using the PRISMA-method for patients with an ED-LOS > 6 hours, excluding children and critical care room presentations.

**Results:**

568 patients visited the ED during the selected week (January 2017). Eighty-four patients (15%) had an ED-LOS > 6 hours and a PRISMA-analysis was performed in 74 (88%) of these patients. 269 root causes were identified, 216 (76%) of which were organisational and 53 (22%) patient or disease related. 207 (94%) of the organisational factors were outside the influence of the ED. Descriptive statistics showed a mean number of 2,5 consultations, 59% hospital admissions or transfers and a mean age of 57 years in the ED-LOS > 6 hours group. For the total group, there was a mean number of 1,9 consultations, 29% hospital admissions or transfers and a mean age of 43 years.

**Conclusions:**

This study showed that the root causes for an increased ED-LOS were mostly organisational and beyond the control of the ED. These results confirm that interventions addressing the complete acute care chain are needed in order to reduce ED-LOS and crowding in ED’s.

## Introduction

Emergency Department (ED) crowding is a serious international health delivery problem [1,2] and it negatively affects the quality and efficiency of ED care [3–6]. ED crowding is defined as “a situation in which the identified need for the emergency service exceeds the available resources for patient care in the ED, hospital, or both” [7].

Prior studies demonstrated that ED crowding is associated with adverse patient outcomes [3–6], including increased mortality [3,5,8]. Furthermore, ED crowding is associated with delays in diagnosis, treatment and hospital admission [3,4] resulting in increased hospital length of stay (LOS) [6], preventable medical errors, increased inpatient costs [3,4] and reduced patient satisfaction and willingness to return [9].

The ED-LOS is the total time from the first documented time after arrival at the ED, whether triage or registration, to the time the patient is discharged from the ED. One of the most commonly reported factors responsible for ED crowding is hospital bed shortage [9,10]. Other reported factors are delays in consultations, radiology, laboratory and treatment by multiple specialties [10,11]. Prior studies investigating the patient related factors contributing to an increased ED-LOS showed that specific subsets of patients are more likely to exceed an ED-LOS > 4 hours [10,12]. These subsets include older patients (65 years and older), patients arriving during peak hours, patients undergoing surgical interventions, neurology or internal medicine patients, patients needing radiology or laboratory testing, and patients categorized as Emergency Severity Index 2 or 3 [10,12].

In the Netherlands, and especially in the Amsterdam region, ED crowding has become an increasing concern over the last few years. In a single quarter of 2015, hospitals in the Amsterdam region had to close their ED’s in over 600 occasions due to crowding [13] implying that the hospitals could no longer guarantee the safe delivery of appropriate care. For example in 2016 the VU University Medical Center (VUMC) in Amsterdam counted 55 ED presentation stops, 175 critical care room stops, 70 thrombolysis stops and 192 acute coronary care stops. From January till September 2017 the VU University Medical Center counted 40 ED presentation stops, 127 critical care room stops, 38 thrombolysis stops and 127 acute heart care stops. Other hospitals in the region showed as many stops or even more. Therefore, health care workers in the Amsterdam region recently even sent a letter of urgency to the Ministry of Public Health asking for help to create remedial measures [13].

Multiple reasons are mentioned to explain the increase in ED crowding in the Netherlands and some of them are specifically related to the organisation of the Dutch health system. In the Netherlands, there is a well-organised primary care system which functions optimally, also during the out of office hours. Most of the patients needing acute care are (initially) seen and treated by the general practitioners [13]. This implies that patients visiting the ED are generally complex; they are older and have more comorbidities than the general population [13]. In addition, recent policy changes with reduction in long-term care facilities have resulted in (frail) elderly people living longer at home. A consequence of this policy is that people still living independently with simple problems, may develop complex care needs unnoticed, because they are not closely monitored in the home-situation. Therefore, patients who are currently visiting the ED are generally older, more frail and sick than those visiting the ED 10 years ago [13].

No in-depth study has yet been performed to systematically analyse the root causes of ED-LOS using a well-established root-cause analysis tool such as the PRISMA-method. The primary aim of this study is to identify the healthcare worker-, organisational-, technical-, disease- and patient- related root causes that may contribute to an increased ED-LOS and formulate recommendations to improve the quality and efficiency of ED care.

## Materials and methods

### Study design and setting

This is an observational record review study focussing on patients visiting the ED of the VU University Medical Center (VUMC) during one busy week in the winter (January 2017). The study was approved by the medical ethics committee of the VUMC. The VUMC is an academic urban level 1 trauma center in Amsterdam, the Netherlands, with 733 beds, approximately 50,000 admissions and 30,000 ED presentations per year. The ED has 27 beds, 4 critical care rooms and 19 treatment rooms. Internal hospital data for the year 2016 indicate that the number of patients visiting the ED per week ranged from 440 to 644 (mean 553) and that, on average, 26% to 29% of the patients visiting the ED were admitted to a hospital. During the study period there were eight residents in emergency medicine, including four fellows of emergency medicine and four non-trainees working in shifts. The supervision of the residents was done by four qualified emergency physicians (EP) and one surgeon. The emergency medicine trainees and EPs belong to the staff of the department of surgery. At the ED of the VUMC referred patients from a general practitioner are seen by residents of various medical specialties under the supervision of medical specialists belonging to the particular department. All self-referrals are seen by the emergency medicine residents and qualified EPs. Depending on the situation and needs of the patient, the EP can consult the medical specialist. If a patient needs more specialised care or needs to be admitted to the ward, the necessary speciality is consulted and the patient is handed over to the specialist for further treatment.

### Data sources

The relevant data was gathered through the Electronic Patient Record (EPR). The EPR systematically stores the nursing, physician, medical specialist, laboratory, radiological and other relevant patient information, including those of the ED stay. When questions remained unanswered or insufficient information could be gathered from the EPR, the physicians or medical specialists involved were interviewed by phone or email to obtain the missing information.

### Data collection and analysis

For each patient visiting the ED during the study week, basic data was gathered from the EPR by two investigators (BR, LV) using a standardized data collection form that was specifically designed for this study. The basic data involved age, gender, date and time of arrival, date and time of ED discharge, discharge destination, triage, number of consultations and the starting and ending speciality.

The triage system used in the VUMC is the “Netherlands Triage System” (NTS) [14]. It is used to assign an urgency code to the patient based on the main complaints, vital functions and severity of symptoms. The NTS consists of 6 categories: U0 through U5, each corresponding to a maximum time to be seen by a physician or medical specialist. In patients triaged U0 and U1 there is a direct life threatening situation, for example a resuscitation or an Airway Breathing Circulation Disability Environment (ABCDE) instable patient, who must be seen immediately. In patients triaged U2 the vital signs are not yet in danger but they are threatened, or they are patients with organ failure, who must be seen as soon as possible, but with a maximum of 10 minutes. Patients triaged U3 are urgent patients and need to be seen within 60 minutes. Patients triaged U4 or U5 are standard and non-urgent patients and need to be seen within 120 minutes and can be handed over to the (out of hours) general practitioner care post which is located next to the ED.

For each patient, the ED-LOS was calculated. Patients were categorized into three groups. The cut-off points for these groups were based on those described in literature for an increased ED-LOS: ED-LOS < 4 hours, ED-LOS 4–6 hours and ED-LOS > 6 hours [1,2,15].

A PRISMA-analysis (Prevention and Recovery Information System for Monitoring and Analysis) was conducted for patients with an ED-LOS > 6 hours. This type of analysis can be used to identify the root causes contributing to an incident. In the analysis, the description of the incident is set at the top of the root causal tree. The incident is then followed by the subsequent identification of the direct and indirect underlying causes [16]. Children (< 18 years) and patients in need of critical care room treatment were excluded from this in-depth analysis.

### PRISMA-analysis

A standardised assessment form that was specifically designed for this PRISMA-analysis was used to collect the relevant data. In order to establish a well-founded root causal profile, the minimum number of patients for evaluation in the PRISMA-analysis was set at 50, which is the generally accepted standard [16]. The data collected for the PRISMA-analysis involved baseline patient and ED admission characteristics.

Direct and indirect causes of an ED-LOS > 6 hours were retrieved by posing the question why the incident, in this case the ED-LOS > 6 hours, has happened. When no further objective causes could be identified, the last indirect cause was considered as the root cause. The PRISMA-analysis also ended when the underlying causes were not related to hospital practices or to any other matters related to hospitalisation.

**Fig 1:**
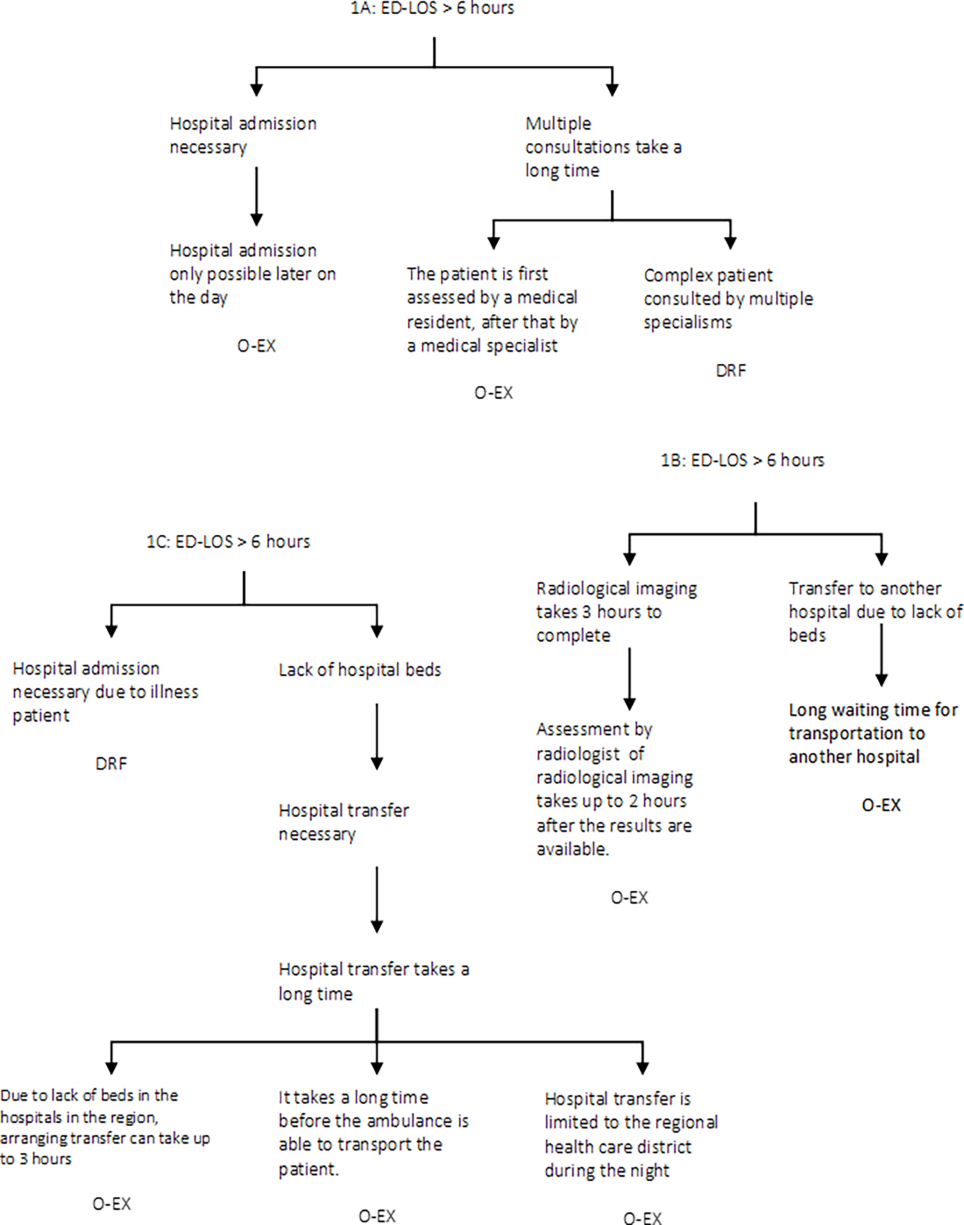
Examples of root causal trees. (a) O-EX External: Organisational External Factor, DRF: Disease Related Factor. (b) O-EX External: Organisational External Factor. (c) O-EX External: Organisational External Factor, DRF: Disease Related Factor.

Root causes were classified using the Eindhoven Classification Model (ECM) [17,18]. This model provides a taxonomy that can be used for the root cause classification in a PRISMA-analysis. The ECM is based on the skill-rules-knowledge-based behavioural model of Rasmussen as well as Reasons’ systemic approach to human error [19–21]. The ECM is also used as a foundational component for the framework of the international classification for patient safety [22,23]. The classification of the root causes according to the ECM is displayed in Table 1 [17,18,24]. In this study, the ECM model was extended with disease related factors following the recommendations of Fluitman et al [25]. Three examples of root causal trees that were identified in this study are displayed in Fig 1.

**Table 1:** Description of categories of the Eindhoven Classification model: PRISMA medical Version [17,18,24].

Main category	Subcategory	Code	Description	Examples (if available)
Technical	External	T-ex	Technical failures beyond the control of the organisation.	Due to a technical failure in the lab, the blood test had to be done again which resulted in a long ED-LOS.
	Design	TD	Failures to poor design of equipment etc.	Not available
	Construction	TC	Correct design inappropriately constructed or placed.	Not available
	Materials	TM	Material defects not classified under TD or TC.	Not available
Organisational	External	O-ex	Failures at an organisational level beyond the control and responsibility of the investigating team.	Patient had to remain in the ED for many hours because the patient could not be admitted due to shortage of available beds in the hospital. Another specialty was called to see the patient and it took more than four hours before the decision for admission was made by the resident and the supervisors of this specialty.
	Transfer of knowledge	OK	Failure resulting from inadequate measures to train or supervise new or inexperienced staff.	Not available
	Protocols	OP	Failures relating to the quality or availability of appropriate protocols.	Not available
	Management priorities	OM	Internal management decisions which reduce focus on patient safety when faced with conflicting priorities.	Because of the crowding in the ED, it takes the ED doctor a long time (1.5hrs) before she can see the patient.
	Culture	OC	Failure due to attitude and approach of the treating organisation.	Within the organisation it is common practice that the patient is sometimes first assessed by a medical student, after that by medical resident and finally by a medical specialist. This causes delays.
Human	External	H-ex	Human failures beyond the control of the organisation/department	There is a changing policy and treatment plan initiated for a patient by a second supervisor in the surgical department.
	Knowledge-based behavior	HKK	Failure of an individual to apply their knowledge to a new clinical situation	Not available
	Qualifications	HRQ	An inappropriately trained individual performing the clinical task	Not available
	Co-ordination	HRC	A lack of task co-ordination within the healthcare team.	A patient has to wait a long time before the consulting medical resident came to see the patient because the ED doctor was late with the consultation request. Long duration before discharge because the IV catheter of the patient still had to be removed.
	Verification	HRV	Failure to correctly check and assess the situation before performing interventions	Not available
	Intervention	HRI	Failure resulting from faulty task planning or performance	Not available
	Monitoring	HRM	Failure to monitor the patient’s progress or condition	The patient remains in the ED longer than needed because the ED doctor took a long time to make the treatment plan.
	Skills-based	HSS	Failure in performance of highly developed skills	Not available
Patient	Patient-related	PRF	Failures related to patient characteristics or conditions, which are beyond the control of staff and influence clinical progress	Patient needed reassurance before discharge which took extra time. It took a long time before the private transport of the patient arrived.
	Disease-related	DRF	Failures related to the natural progress of disease which are beyond control of patient, its carers and staff	The patient had very complex problems which resulted in many additional diagnostic tests.
X	Unclassifiable	X		Not available

### Classification

The data were independently assessed by two medically and PRISMA-trained investigators (BVR, LV). The root causal trees of both investigators were compared and discussed with a third PRISMA-trained investigator (HM) until consensus was reached.

In order to draw conclusions on probable remedial measures which can be implemented in or outside the ED, it was decided that all technical, organisational and human factors outside the control of the ED itself were considered as external factors. When the difference between internal and external, or between human, technical or organisational factors was subject to debate, two other experts involved in ED patient care, the director of the ED residency program (PN) and an experienced emergency physician (BD) were asked for advice. It was decided that root causes that occurred frequently (when a structural pattern was seen) were considered organisational.

Each structurally occurring root cause was further investigated by contacting the involved department. It was investigated whether the identified cause also occurred frequently at hospital or departmental level, or whether it was an incident, for example due to the behaviour of an individual person. Structurally occurring root causes were for example long door to -medical specialist time and long radiological waiting and evaluating time. Both causes were classified as external organisational factors due to their structural occurrence.

### Statistical analysis

IBM SPSS Statistics, Chicago, USA, Version 23.0 was used to calculate the descriptive characteristics and frequencies. Outcome measures are presented as frequencies and percentages of the total number of patients per category.

## Results

### Patient characteristics

568 patients visited the ED during the selected one-week period (Table 2). 215 patients (38%) had an ED-LOS > 4 hours, of which 86 patients (40%) had an ED-LOS > 6 hours. Patients with an ED-LOS > 6 hours more frequently visited the ED on Monday, Thursday and Friday. Most patients visited the ED during the afternoon and evening.

**Table 2:** Patient characteristics and distribution of visiting patients over the week.

	Patients visiting emergency department (ED) (n, %)			
	**Less than 4Hrs.**	**4 to 6 Hrs.**	**More than 6Hrs.**	**All**
**Male**	184 (52,1)	62 (48,1)	44 (51,2)	290 (51,1)
**Median age in years [range, IQR]**	32 [0–99, 42]	54 [0–94, 43]	58 [9–97, 39]	42 [0–99, 44]
**Frequency per stage of life**				
*<18 years*	93 (26,3)	11 (8,5)	3 (3,5)	107 (18,8)
*18–40 years*	115 (32,6)	34 (26,4)	19 (22,1)	168 (29,6)
*40–65 years*	80 (22,7)	37 (28,7)	28 (32,6)	145 (25,5)
*>65 years*	65 (18,4)	47 (36,4)	36 (41,9)	148 (26,1)
	**Less than 4 Hrs.**	**4 to 6 Hrs.**	**More than 6 Hrs.**	**All**
**Patients**	353 (62,1)	129 (22,7)	86 (15,2)	568 (100)
*Monday*	44 (53,7)	22 (26,8)	16 (19,5)	82 (100)
*Tuesday*	48 (66,7)	16 (22.2)	8 (11,1)	72 (100)
*Wednesday*	58 (67,4)	21 (24,4)	7 (8,1)	86 (100)
*Thursday*	51 (61,4)	14 (16,9)	18 (21,7)	83 (100)
*Friday*	52 (64,9)	15 (18,5)	14 (17,3)	81 (100)
*Saturday*	50 (55,6)	28 (31,1)	12 (13,3)	90 (100)
*Sunday*	50 (67,6)	13 (17,6)	11 (14,9)	74 (100)

### Patient flow through the emergency department

The number of patients visiting the ED in the selected week was slightly more than an average week in 2016 (mean 553 patients; range 440–644). Patients with an ED-LOS < 4 hours were mostly triaged as U3 (36%) and U5 (26%). Patients with an ED-LOS > 4 hours and ED-LOS > 6 hours were most likely to be triaged as U2 (33% /30%) or U3 (40% /34%) (Table 3).

**Table 3:** Flow of patients through the ED.

	Patients visiting emergency department (ED) (n, %)			
	Less than 4 Hrs.	4 to 6 Hrs.	More than 6 Hrs.	All
**Patients**	353 (100)	129 (100)	86 (100)	568 (100)
**Triage**				
*U0*	3 (0,8)	1 (0,8)	1 (1,2)	5 (0,9)
*U1*	27 (7,6)	13 (10,1)	9 (10,5)	49 (8,6)
*U2*	67 (19,0)	44 (34,1)	26 (30,2)	137 (24,1)
*U3*	127 (36,0)	40 (31,0)	34 (39,5)	201 (35,4)
*U4*	27 (7,6)	7 (5,4)	3 (3,5)	37 (6,5)
*U5*	92 (26,1)	24 (18,6)	13 (15,1)	129 (22,7)
*Unknown*	10 (2,8)	0 (0,0)	0 (0,0)	10 (1,8)
**Starting specialism**				
*Emergency medicine*	200 (56,7)	42 (32,6)	28 (32,6)	270 (47,5)
*Cardiology*	8 (2,3)	1 (0,8)	0 (0,0)	9 (1,6)
*Internal medicine*	34 (9,6)	43 (33,3)	34 (39,5)	111 (19,5)
*Neurology*	18 (5,1)	11 (8,5)	5 (5,8)	34 (6,0)
*Paediatrics*	44 (12,5)	9 (7,0)	0 (0,0)	53 (9,3)
*Surgery*	16 (4,5)	14 (10,9)	10 (11,6)	40 (7,0)
*Urology*	12 (3,4)	1 (0,8)	3 (3,5)	16 (2,8)
*Other*	21 (5,9)	8 (6,2)	6 (7,0)	35 (6,1)
**Ending specialism**				
*Emergency medicine*	197 (55,8)	41 (31,8)	22 (25,6)	260 (45,8)
*Cardiology*	9 (2,5)	2 (1,6)	1 (1,2)	12 (2,1)
*Internal medicine*	35 (9,9)	37 (31,9)	42 (48,8)	114 (20,1)
*Neurology*	18 (5,1)	13 (10,1)	8 (9,3)	39 (6,9)
*Paediatrics*	44 (12,5)	9 (7,0)	0 (0,0)	53 (9,3)
*Surgery*	17 (4,8)	10 (8,6)	12 (13,9)	39 (6,9)
*Urology*	12 (3,4)	1 (0,8)	4 (4,7)	17 (3,0)
*Other*	21 (5,9)	8 (6,9)	5 (5,1)	34 (6,0)
**Discharge destination**				
*Discharge directly from the ED*	297 (84,1)	67 (51,9)	34 (39,5)	398 (70,1)
*Hospital admission*	52 (14,7)	52 (40,3)	40 (46,5)	144 (25,4)
*Transfer to another hospital due to lack of beds*	4 (1,1)	8 (6,2)	11 (12,8)	23 (4,0)
*Deceased*	0 (0,0)	2 (1,6)	1 (1,2)	3 (0,5)
**Mean number of consultations (SD)**	1,6 (0,6)	2,3 (0,6)	2,5 (0,8)	1,9 (0,8)

Patients in the total population were most often treated by emergency physicians (46%). The average number of consultations for this group was 1,9, the percentage of hospital admissions or transfers to other hospitals 29%, and the percentage of patients discharged home 70%. Patients with an ED-LOS > 6 hours were most often treated by internal medicine (49%) as the primary speciality. The average number of consultations was 2,5, the percentage of hospital admissions or transfers 59%, and the percentage of patients discharged home 40%.

### PRISMA-analysis

Seventy-four patients were eligible for inclusion in the PRISMA-analysis. Forty of these patients (54%) were undergoing outpatient treatment by a specialist during the year preceding the ED visit, 36 patients (49%) had polypharmacy (more than 5 drugs) and 34 patients (49%) had been hospitalised within the year prior to the ED visit.

Of the 74 patients, 22 patients (30%) received medical services by the hospital 30 days prior to this ED presentation. 11 of these 22 patients (15%) were re-presentations at the ED, meaning they visited the ED in the 30 days prior to this ED visit. All 11 patients presented with the same complaint as their prior ED visit. Eight of these 11 patients were discharged home, 3 of these 11 patients were admitted to the hospital after the ED re-presentation. The remaining 11 patients had been admitted to the hospital in the last 30 days prior to this ED presentation.

For the PRISMA-analysis, 17 patient records were incomplete and in need of additional information. For example, in some cases it was unclear how long it took to arrange admission to the hospital or a transfer to another hospital, what the cause of the delay was in seeing the patient by the specialist, or why discharge from the ED took a long time. In 13 out of these 17 cases, additional information could be gathered by contacting the specialist or nurse in charge. This information was collected in a generic, anonymous form. In each case, the information obtained was of added value, resulting in a more complete and reliable root causal tree.

**Fig 2:**
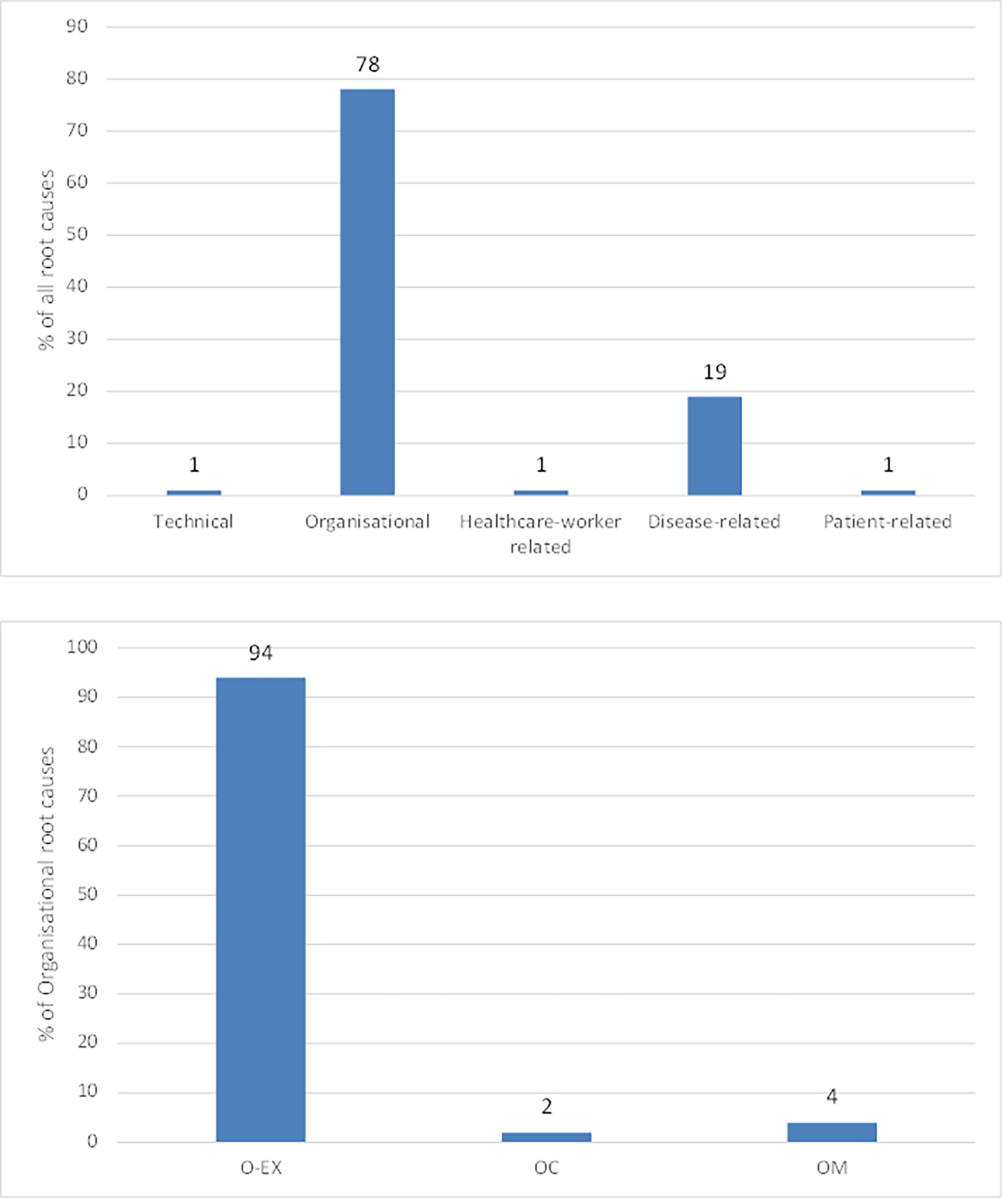
(a) Main categories of root causes. (b) Distribution of organisational root causes.

In total, 276 root causes were identified for 74 PRISMA-analysed patients with an ED-LOS > 6 hours. Three patients (4%) with an ED-LOS > 6 hours had one root cause, 12 (16%) patients two, 15 (20%) patients three, 24 (33%) patients four, 12 (16%) patients five and 8 (11%) patients six or more. The 276 root causes could be categorised into 86 different unique causes. The distribution and classification of the 276 root causes using the extended ECM-model is displayed in Fig 2A.

216 (78%) of all identified root causes were organisational. The causes describe failures at an organisational level, such as management priorities, organisational culture, and the quality and availability of protocols. 203 (95%) of these organisational root causes were outside the influence of the ED, they are categorised as external organisational causes (Fig 2B). Thirty-three (15%) of these external causes were related to transfer to other hospitals, 44 (21%) to a delay in hospital admission. For 50 (23%) root causes it was not exactly clear what caused the delay. 25 (12%) root causes were due to a delay between consultations of different medical specialties and 22 (10%) root causes were due to a long door- to- medical specialist time because a medical student was the first assessor of the patient. 41 (19%) root causes were due to long radiological waiting and evaluation times.

Fifty-three (19%) of all the identified root causes (n = 276) were disease related. For example, because of the severity or progression of a disease. Other types of root causes occurred less frequently. These included healthcare worker related factors (n = 4, 1%) such as forgetting to remove an IV drip of a patient being discharged; technical factors (n = 2, 1%), such as the need for repeated diagnostics due to blood being haemolytic; and patient related factors (n = 2, 1%) such as patients requesting to be transferred to another hospital.

## Discussion

This is the first study to systematically investigate the root causes for an ED-LOS > 6 hours in the Netherlands using PRISMA-analysis. The data suggests that patients who exceeded an ED-LOS > 6 hours were generally more complex and older. The majority of root causes contributing to an increased ED-LOS were related to organisational factors such as shortage of beds leading to hospital transfer, radiological imaging or sequential specialist consultations. Most of these organisational causes were classified as external as they were due to factors outside the control of the ED. This implies that measures implemented with the aim of improving ED-LOS should cover the complete acute healthcare chain rather than the ED only.

The results of this study are consistent with those of other authors. Van der Linden et al. suggested that additional medical staff during peak hours decreased the need for ancillary diagnostics and shortened the waiting time for contact with the residents or medical specialists. They also demonstrated a positive impact on the ED-LOS and patient satisfaction [26]. In addition, a prospective study in the Netherlands from Vegting et al. concluded that factors which lead to ED stagnation were old age, treatment by more than one speciality during ED stay, and undergoing radiological testing [10]. Moreover, in another study by Van der Linden et al. laboratory and radiology delays, consultation delays, and hospital bed shortages for patients in need of hospital admission were the most important factors contributing to ED crowding [12]. Furthermore, the results are consistent with those of Pines et al. who identified that the main cause for crowding was the boarding of patients from the ED [2].

Finally, our results show that 16 out of the 86 patients with an ED-LOS of more than 6 hours, are triaged U4 of U5. 38% of these patients were admitted to the hospital. This may indicate that the NTS may has undertriaged these patients and therefore the patients did not receive timely care. Byrne et al. derived an Acute Illness Score, based on laboratory data [27]. This is an age-adjusted risk estimator to predict the 30-day in-hospital mortality. They hypothesise that a high Acute Illness Score is the most significant determinant of an emergency admission. Since we have a significant part of patients with a low triage code with an ED-LOS of more than 6 hours some of whom in addition also required hospital admission, it may be advisable to introduce the Acute Illness Score on top of NTS to recognize patients with a low triage code in need of admission.

This PRISMA-analysis was conducted according to established standards. The one-week period was sufficient to meet the minimum requirements for a root-cause profile (n = 50) [16]. Basic demographic data was gathered for all patients visiting the ED rather than only for those subject to the PRISMA-analysis in order to provide some background information for the situation at the ED during the study period.

Although the study was conducted in the Netherlands, the results can also be of interest to other countries struggling with the same problem. However, the differences in the national organisation of healthcare should be taken into account. In the Netherlands, the organisation of healthcare differs from the rest of Europe. Instead of patients visiting the ED directly, it is standard practice that patients will first visit or consult a general practitioner (GP) 24x7 hours with any complaint. GPs treat simple injuries, non-acute and non-life threatening complaints. In addition, they assess whether they can treat the patient or whether referral to other, more specialised, health care professionals is warranted. This means that the GP functions as a gatekeeper for secondary healthcare services as e.g. hospital care, specialist psychological counselling and rehabilitation. In addition, we can hypothesise that patients visiting the ED in the Netherlands are generally more complex than patients visiting the ED in other countries without such a strong primary care system.

This study has some limitations. Firstly, the analysis depends on the correct and complete entry of data by the health care workers in the EPR. In order to retrieve the missing information, the relevant physicians or medical specialists were interviewed. Secondly, the study evaluated a one-week period in a single academic centre in the Netherlands in the winter period (one of the busiest period in the hospital). It should be acknowledged that this week may not be representative for other seasons, as for example, it is known that patients are more likely to suffer from influenza in the winter compared to the summer, but on the contrary, less likely to have dehydration [28,29]. Moreover, in the selected week there was an increased flow of patients in the ED`s around Amsterdam, leading to frequent ED- admission stops in several hospitals nearby. This may have increased the ED-LOS in the study hospital because the transfers to other hospitals were difficult or almost impossible. All this may have contributed to an increased ED-LOS (ED crowding) in our hospital.

### Recommendations

The findings of this study can be used to develop a variety of measures to avoid increased ED-LOS and ED-crowding. The following recommendations are proposed.

ED physicians and medical specialists should be instructed to monitor the time a patient is staying at the ED more closely so that any increased ED-LOS in a given patient will be observed and remedial measures taken. In addition, they could be instructed to monitor ED-crowding through checklists such as mEDWIN or NEDOCS [30,31].

In addition, the added value of patient assessment by teams rather than individuals could be considered by hospital management, especially for patients in need of complex care, or in need of treatment by multiple specialities [32]. In the last years an increasing number of elderly patients with polypharmacy and multi-morbidity present to the ED in need of complex and multidisciplinary care. In order to further increase efficiency, the authors consider that triage in these elderly patients with complex care needs can best be done by experienced physicians rather than young inexperienced doctors or nurses. In addition, stimulating team work from all specialities involved in caring for these patients in the acute care chain will improve the patient flow and the quality of patient care [32]. For example, Vegting et al. suggested that improving the coordination of care would speed up decisions making, leading to a shorter completion time for patients [11].

Moreover, decision makers could pay increased attention to the need for additional lack of human capacity or radiological imaging and support ad-hoc or more permanent measures to fill the gap. All stakeholders should duly consider the added value of remedial measures identified by other authors. Richardson et al. found that teaming ED medical staff, reorganising bed flow, appointing a long LOS committee and setting an ED navigator role significantly improved ED flow, which is likely to reduce crowding [14].

The introduction of Acute Medical Units is a complementing concept to stimulate multidisciplinary teamwork and improve patient flow in order to reduce ED-LOS. Rombach et al. showed that the implementation of the Acute Medical Unit in the VUMC resulted in an increase in the total number of admissions, decrease in average lengths of hospital stay on normal care departments, decrease in number of admission refusals and a decrease in the number of readmissions[33].

In addition, specialties could develop specific wards for more complex and specialised ED presentations. For example, Balakrishnan et al. evaluated the implementation of an Acute Medical Unit in Singapore, where only patients who were suspected to have an infection relation condition were admitted from the ED [34]. The implementation of this ward resulted in a decrease in readmission within 15 and 30 days and a decrease in hospital LOS. This may also be illustrated by strategies to improve the care of acutely unwell cancer patients in the United Kingdom. The implementation of a specialist hotline accessible for patients, an inpatient clinic/chemotherapy/radiotherapy and the implementation of a cancer assessment unit are examples of models to improve emergency care for specific and complex specialised emergency presentations [35].

In any case, it should be ensured that immediate access to ambulance transportation would remain available for patients in need of hospital transfers.

Finally, an increase in the number of hospital beds could be considered together with a regional system indicating bed availability, as this would avoid delays in admission or transfer.

More insight into the patient pathways before admission to the ED could be useful to develop remedial measures directed at preventing ED presentations. This would be especially interesting for the more complex and generally older patients, since they are more likely to have an increased ED-LOS. For example, on a patient level, admission to the ED may be due to a lack of adherence to drug treatment [36]. Such in-adherence could be due to the inability of the patient to administer the product itself in combination with the lack of timely caregiver assistance.

## Conclusion

This study shows that increased ED-LOS was mainly due to organisational factors outside of the influence of the ED. Therefore interventions targeting the complete acute care chain are needed. Further research is needed to gather insight into the causes within the complete care chain in order to decrease ED-LOS or prevent ED presentations.

## Supporting information

S1 Dataset Patient characteristics and information from the EPR from all patients (n = 568) visiting the ED during the selected one-week period. (SAV)
